# The Characterization of a Low-Calorie and Lactose-Free Brown Fermented Milk by the Hydrolysis of Different Enzymatic Lactose

**DOI:** 10.3390/foods13182861

**Published:** 2024-09-10

**Authors:** Han Tao, Shuo-Qian Li, Meng-Jia Fang, Wan-Hao Cai, Song Zhang, Hui-Li Wang

**Affiliations:** 1 Engineering Research Center of Bio-Process, Ministry of Education, Hefei University of Technology, 193 Tunxi Road, Hefei 230009, China; nancyfoodscience@hotmail.com (H.T.); lishuoqian1230@163.com (S.-Q.L.); sunflowerjia@foxmail.com (M.-J.F.); wanghl@hfut.edu.cn (H.-L.W.); 2 School of Food Science and Engineering, Hefei University of Technology, Hefei 230009, China; 3 Department of Food Science and Engineering, Moutai Institute, Renhuai 564502, China

**Keywords:** lactose-free, brown fermented milk, storage stability, low calorie, harmful Maillard reaction products

## Abstract

The adoption of brown fermented milk in the normal diet and daily beverages is accompanied by significant sugar intake and a high public health burden. To reduce the sugar content in dairy products while maintaining optimal nutritional properties, a novel low-calorie, lactose-free brown fermented milk was developed through enzymatic hydrolysis and the Maillard reaction. The optimal product was achieved using low-temperature lactase, where the lactose and glucose content were reduced 33-fold and 2.4-fold to 0.06 g/100 g and 13.32 g/L, respectively, meeting the criteria for being lactose-free (<0.5 g/100 g). Meanwhile, hazardous compounds such as 5-hydroxymethylfurfural and 3-deoxyglucosone were reduced by more than 20%. After 28 days of storage, the water-holding capacity and suspension stability remained notably stable, and the protein composition was also more enriched compared to commercial milk. It is expected that this low-calorie dairy product may promote growth in the dairy market.

## 1. Introduction

Brown fermented milk is a type of fermented milk characterized by its dark brown color, intense aroma, and velvety mouthfeel. These unique organoleptic properties originate from the Maillard reaction, which involves the interaction between reducing sugars and proteins present in milk [1]. Brown fermented milk is mainly popular in China and Russia and comes in various types of products, such as cooked and chargrilled fermented milk [2]. In recent years, it has also gradually entered the East Asian and Western markets, with Yakult’s products an example, showing continuous growth each year [3]. According to reports, many dairy companies produce brown fermented milk in the millions of tons annually [4,5]. Considering the extensive free market globally, brown fermented milk has significant potential for development and economic benefits.

Milk has become a staple in the normal diet of most countries [6]. Due to the novel and unique flavor of brown fermented milk, it not only currently appears in the normal diet but is also becoming popular as a daily beverage [7]. However, the currently available brown fermented milk in the market is produced by adding exogenous reducing sugars to raw milk [8]. Therefore, consuming the brown milk as a part of the normal diet over a long period can result in a significant intake of sugar and lead to a high public health burden [9]. Many countries have already begun proposing macro policies to address the challenge. For instance, China’s “Healthy China Action” (2019–2030) strongly promotes the use of sugar-reduced foods [10]. This has led to a growing demand from both the industry and consumers for sugar-reduced products. At the same time, the Maillard reaction produces hazardous chemicals such as 3-deoxyglucosalone and 5-hydroxymethylfurfural as the by-products of sugar degradation due to high-temperature processing and prolonged storage conditions, which have carcinogenic and genotoxic effects on animal and human cells and can cause diseases including diabetes and neurological diseases [11]. Another concern is that variations in protein composition can have a substantial impact on nutrition and health. It is known that critical proteins and amino acids are essential for body development, tissue growth, and cell function; some proteins can further function as hormones or antibacterial agents [12]. Since the protein composition in brown fermented milk may differ from that of regular milk due to the starter strain and the additives, the uncertainty regarding its nutritional value is much greater [13]. Other concerns include the presence of lactose which is problematic for individuals with lactose intolerance and so on [14]. Enzymatic hydrolysis of lactose is a widely used process for reducing lactose content [15]. The process of lactose hydrolysis does minimal damage to the nutritional components in milk and is highly specific. The optimal pH and temperature of commercial β-galactosidases are 7.0 and 35 to 40 °C, which are susceptible to result in the contamination of milk [16]. Thermostable and cold-adapted β-galactosidases had significant advantages in processing, such as a higher substrate solubility and reaction rate, as well as a lower probability of microbial contamination [17]. All these concerns hinder the widespread promotion of brown fermented milk in the commercial market. Thus, there is an urgent need to develop a brown fermented milk with low sugar content.

Given these circumstances, there is an urgent need to develop low-sugar and lactose-free brown healthy milk and comprehensively evaluate its protein nutritional quality and safety [18]. However, research and development on lactose-free brown fermented dairy products is still limited, lacking investigation into optimizing production processes, conducting sensory evaluations, assessing the storage stability, and assessing the protein functional properties of such lactose-free brown dairy products.

In this study, a low-calorie and lactose-free brown fermented milk was developed by the hydrolysis of different enzymatic lactose. The physicochemical properties were characterized and proteomic analyses of these lactose-free brown milk were performed, providing a comprehensive evaluation of their nutrition, functionality, and safety. It is anticipated that this novel dairy product will broaden the fermented milk market and promote the development of the dairy industry.

## 2. Materials and Methods

**Materials**: Commercial fresh milk (Inner Mongolia Yili Industrial Group Co., Ltd., Hohhot, China) was chosen as the raw milk for fermentation. A commercial freeze-dried starter culture consisting of *Streptococcus thermophilus* and *Lactobacillus bulgaricus* was supplied by Angel Yeast Co., Ltd (Yichang, China). Commercially available fermented milk was purchased from Hefei Hongfu Supermarket and contained added sugars and stabilizers (ingredient list: raw milk, white granulated sugar, edible glucose, whey protein powder, Streptococcus thermophilus, Lactobacillus bulgaricus, Lactobacillus paracasei, hydroxypropyl distarch phosphate, monodiglyceride diacetyltartrate, pectin). Medium-temperature lactase (7500 U/g) and low-temperature lactase (7000 U/g) were obtained from Novozymes (Denmark) and DSM (UK), respectively. Standards including 5-Hydroxymethylfurfural (5-HMF, 98%) and 3-Deoxyglucosone(3-DG,98%) were obtained from Sigma (Taufkirchen, Germany). O-phenylenediamine (OPD) was purchased from Shanghai Yuanye Bio-Technology Co., Ltd. (Shanghai, China). Acetic acid, methanol, and acetonitrile (HPLC-grade) were purchased from Solarbio Chemicals (Beijing, China). A Thermo Genpure UF system was used to prepare ultrapure water. All other chemicals and regents used were of analytical grade.

**The preparation of lactose-free brown fermented milk:** All the fermented milk used in this study was produced using small production line equipment available at the school level, and was autoclaved and sterilized prior to use to ensure sterility throughout the production process. The fresh milk containing high-quality protein underwent homogenization and pasteurization at a homogenization pressure of 18–20 MPa and a sterilization temperature of 95 ± 2 °C for 300–400 s. Next, low-temperature-type lactase, at 0.05–0.09% relative to the weight of the milk, was added into the milk then hydrolyzed at 12 ± 1 °C for 15–18 h, while for medium-temperature-type lactase, 0.60–0.80% was added into the milk then hydrolyzed at 42 ± 1 °C for 2–3 h. Then, the respective mixtures were heated to 95 ± 2 °C for browning for 2–3 h. The product was then cooled down to 42 ± 1 °C, and a fermentation agent was inoculated under sterile conditions with a feed weight of 0.15%. The fermentation was maintained at 42 ± 1 °C for 6–8 h while the agitator intermittently ran at 60% speed for 20 min at a time. The fermentation process was halted when the milk reached a pH of 4.5–4.7 and a viscosity of ≥900 mPa·s. After that, the temperature was quickly dropped to between 20 and 25 °C. The material was then rolled up and down in a clockwise direction using a blender. Brown fermented milk was achieved by stirring for 10 min at 100% rotating speed, achieving a uniform surface without apparent clots, followed by refrigeration at 4–6 °C for 16–24 h. The yogurt samples were prepared in triplicate and stored at 4 °C for 0, 7, 14, 21, and 28 days.

**The detection of sugar and hazardous substance content:** The content of lactose was obtained by using the lactose test box (Rising Biology) method. In brief, 0.2 g of the yogurt was taken and diluted 100 times. After centrifugation at 12,000 rpm for 10 min, 10 μL of supernatant was transferred to a 2 mL EP tube. Then, different ratios of reagents in the test box were added to the sample. The mixture was incubated for 30 min in the dark at 37 °C. The absorbance was measured at a wavelength of 510 nm and then converted to lactose content according to the instruction manual.

The content of glucose was determined by using the glucose text box (Beijing Solarbio Science & Technology Co., Ltd., Beijing, China) method. An amount of 0.1 g of yogurt was homogenized with 20 mL of ultrapure water. Centrifugation was then carried out for 10 min at 8000 rpm. Then, different ratios of reagents in the test box were added to the volume of 100 μL of the supernatant. After mixing, test tubes were incubated for 15 min at 37 °C. The absorbance was measured at a wavelength of 505 nm. The glucose content was calculated by the formula in the instruction manual.

5-Hydroxymethylfurfural content was determined by HPLC following the method of Li et al. [19]. Briefly, 10.00 g of sample was mixed with 5 mL of 0.15 M oxalic acid. Then, the sample was transferred into a 50 mL volumetric flask and 3 mL of potassium ferrocyanide solution (0.25 M) and 3 mL of 1 M zinc acetate solution was added; the volume was made up to 50 mL with methanol. The solution was centrifuged (4000 rpm, 10 min), evaporated to 5 mL, and filtered (0.45 μm). The HPLC analysis was conducted using a Waters 2695 system coupled with a Waters 2998 ultraviolet detector and an Xbridge C18 (5 μm) column (4.6 mm × 250 mm) (Waters, Milford, MA, USA). Detection was set at 280 nm. The column temperature was 30 °C and the injection volume was 10 μL. Methanol and water (85:15, *v*/*v*) were used as the mobile phase for isocratic elution at a flow rate of 1 mL/min.

3-Deoxyglucosone was analyzed by HPLC system according to a previously published method of Aktağ et al. [20], with some modifications. In brief, 1.0 mL of each sample was mixed with 70 µL of ortho-phenylenediamine (60 N) and reacted in a water bath for 30 min at 60 °C, and then filtered by a 0.45 µm aqueous microporous filter membrane. The separation of the compounds was carried out at 25 °C with a flow rate of 0.8 mL/min by using a Waters Symmetry C18 (5 μm) column (4.6 mm × 250 mm). Detection was set at 313 nm. The injection volume was 20 μL and the mobile phase consisted of 0.15% acetic acid aqueous solution (A) and acetonitrile (B). The gradient elution procedure was as follows: 0–10 min (92→60% A); 10–12 min (60→52% A); 12–13 min (52→40% A); 13–15.5 min (40→20% A); 15.5–20.5 min (20% A); 20.5–25.5 min (20→55% A), 25.5–30.5 min (55→92% A).

**Whey rate:** Whey rate was measured according to the modified method given by Qing et al. [21]. A yogurt sample (50 g) was centrifuged at 100 rpm for 1 min. The yogurt sample sat for 24 h, then container weight (*W*_0_), the weight of the fermented milk and the container (*W*_1_), and the weight of the fermented milk and container after removing the supernatant (*W*_2_) were measured to calculate the whey rate, where

(1) $$\mathrm{Whey}\;\mathrm{Rate}=\frac{W_{1}-W_{2}}{W_{1}-W_{0}}\times 100\%$$

**Water-holding capacity (WHC):** The experimental conditions were outlined by He et al. [22]. An amount of 20 g of prepared yogurt was centrifuged for 15 min (3500 rpm, 25 °C) before discarding the supernatants. The water-holding capacity of the prepared sample was calculated using the following formula:

(2) $$WHC\%=\frac{W_{1}}{W_{2}}\times 100\%$$

where *W*_1_ is the sediment weight, and *W*_2_ is the sample weight.

**Suspension stability:** According to Li et al. [23], 1 g of the yogurt was taken and diluted 80 times. At a wavelength of 540 nm, the absorbance of the measured sample was denoted as *A*_1_. Then, 50 mL of the diluted sample was extracted at 4000 rpm, centrifuged for 10 min, and the upper layer of emulsion was taken to measure the absorbance at the wavelength of 540 nm, which was recorded as *A*_2_. The suspension stability was calculated according to Formula (3). The larger the R value, the better the suspension stability of the sour milk sample.

(3) $$R\%=\frac{A_{2}}{A_{1}}\times 100\%$$

where *R* is yogurt stability; *A*_1_ is the sample absorbance; and *A*_2_ is the absorbance after centrifugation.

**Surface topography:** Milk samples were initially stored at −80 °C for 24 h in a refrigerator, followed by freeze-drying using a FD-1A-50 freeze dryer (Bo Yi Kang Biotechnology Co., Ltd., Beijing, China). The lyophilized samples were then affixed to aluminum columns using double-sided tape, and subsequently coated with a thin gold layer. To render the samples electrically conductive, a palladium alloy was deposited onto the aluminum columns through ion sputtering [24]. Thereafter, they were positioned within a high-vacuum cryo-scanning electron microscope (COMEX EM30+, Taejon, Korea) for microstructural observation. The images were recorded at different magnifications for better evaluation.

**Rheological characterization.:** The rheological properties of the yogurt samples were evaluated by an Anton-Paar MCR502 Rheometer (Graz, Austria) with a 25 mm diameter parallel plate. A quantity of 1 mL of emulsion was carefully placed on the rheometer’s plateau for 5 min equilibration prior to measurement. Continuous flow measurements were performed by incrementally increasing the shear rate from 0.1 s^−1^–100 s^−1^. The obtained points were subjected to rSpace software for the analyses of shear stress, shear rate, and apparent viscosity [25].

**Protein extraction:** The protein extraction samples were immediately immersed in an ice-water bath to facilitate rapid freezing. Protein precipitation was subsequently carried out by adding five volumes of ice-cold acetone to a 200 μL aliquot of the sample. The mixture was then centrifuged at 4 °C for 10 min at 7000 rpm to pellet the precipitated proteins, and the supernatant was carefully removed. A volume of 300 μL of lysis buffer was added to the precipitated protein and incubated in an ice-water bath for 20 min to facilitate protein solubilization. Finally, the solubilized protein was recovered by centrifugation at 4 °C for 10 min at 12,000 rpm, and the supernatant containing the soluble protein fraction was collected for further treatment [26].

**Reduction and alkylation:** The extracted proteins were incubated in 5 mM dithiothreitol (DTT) solution at 55 °C for 5 min to reduce disulfide bonds. Then, the sample was brought to ambient temperature by introducing 15 mM iodoacetamide (IAA) for 30 min, during which the reduced disulfide bonds (i.e., thiol bond) were alkylated to prevent the reformation of disulfide bonds.

**Protein digestion:** Trypsin was solubilized in a resuspension buffer to attain a concentration of 0.5 μg/μL and incubated at ambient temperature for 5 min to ensure complete dissolution. Then, the protein sample and trypsin solution were comprehensively mixed, maintaining a trypsin-to-protein ratio of 1:50 (*w*/*w*). Following a brief centrifugation step, the mixture was subjected to overnight incubation at 1000 rpm and 37 °C to facilitate enzymatic digestion. Trifluoroacetic acid (TFA) was subsequently used to acidify the resultant peptide mixture, with the final TFA concentration being adjusted to 0.5% to terminate the digestion reaction.

**Peptide desalting:** For column activation, 400 μL of Buffer B (0.1% formic acid, 80% acetonitrile) was dispensed into the de-mineralized C18 spin column. Centrifugation was performed at 2000 rpm for 1 min to ensure the entire solution flowed gradually through the column bed. Subsequently, 400 μL of Buffer A (0.1% formic acid, 2% acetonitrile) was added for column equilibration, followed by centrifugation at 2000 rpm for 1 min, allowing the solution to traverse the column slowly. The sample supernatant containing the peptide mixture was then loaded onto the column and centrifuged at 2000 rpm for 1 min to facilitate the binding of the peptides to the stationary phase. Two washing steps were performed by adding 400 μL of Buffer A, centrifuging at 2000 rpm for 1 min, and allowing the wash solution to elute gradually from the column, effectively removing unbound contaminants. The desalted peptides were then eluted with 200 μL of Buffer B and collected in a fresh microcentrifuge tube. The eluate was subsequently dried by vacuum centrifugation overnight at 4 °C. For mass spectrometric analysis, the dried peptides were reconstituted in 100 μL of mobile phase A, and the supernatant was recovered by centrifugation to remove any insoluble particulates.

**Nano LC-MS/MS analysis:** For each sample, 200 ng of the total peptide mixture was subjected to separation and analysis using a nano-UPLC system (nanoElute2) coupled to a timsTOF Pro2 mass spectrometer (MS, Bruker, Germany) equipped with a nano-electrospray ionization source. Chromatographic separation was achieved on a reversed-phase column (PePSep C18, 1.9 μm, 75 μm × 25 cm, Bruker, Germany) employing mobile phases consisting of water with 0.1% formic acid (phase A) and acetonitrile with 0.1% formic acid (phase B). The peptides were eluted using a 60 min gradient at a flow rate of 300 nL/min, with the following gradient profile for solvent B: 2% for 0 min, 5–22% for 45 min, 22–37% for 5 min, 37–80% for 5 min, and 80% for 5 min. The mass spectrometer was operated in a data-dependent acquisition mode, employing the Parallel Accumulation-Serial Fragmentation (PASEF) technique. The scan range was set from 100 to 1700 *m*/*z*. During PASEF MS/MS scanning, the collision energy was linearly ramped up according to the ion mobility, ranging from 20 to 59 eV (1/K_0_ = 0.6–1.6 V s/cm^2^).

**Protein identification and quantification:** Raw MS files were processed using SpectroMine software (version 4.1.230421.52329). MS/MS spectral libraries were then searched against species-specific UniProt FASTA protein databases. The database used for each sample corresponded to the organism of origin (e.g., uniprot-Bos taurus (Bovine)-9913-2020-10.fasta for bovine samples). The carbamidomethylation of cysteine residues was set as a fixed modification, while the oxidation of methionine residues and N-terminal protein acetylation were considered as variable modifications. Trypsin was specified as the digestion enzyme, allowing for a maximum of two missed cleavages. To minimize false positives, a stringent false discovery rate of 1% was applied at both the peptide spectrum match and peptide levels. The initial precursor mass tolerance for peptide identifications was set to 10 ppm. Both unique and razor peptides were used for protein quantification, with the quanitty of total peptide employed for normalization.

**Statistical analysis:** All data were obtained in at least triplicate and expressed as mean ± standard deviation. Statistical analysis was processed by the analysis of variance (ANOVA) with the software SPSS 25 (SPSS Institute Inc., Cary, NC, USA). Significant differences were detected by Tukey tests (*p* < 0.05).

## 3. Results and Discussion

**Figure 1:**
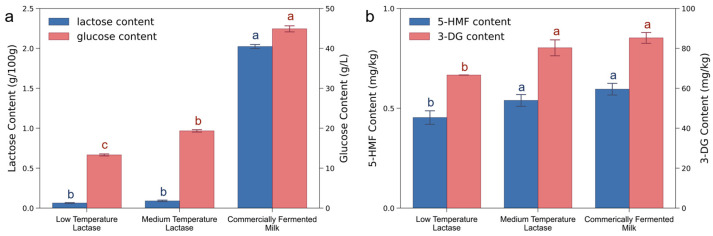
Comparison of (**a**) lactose and glucose contents and (**b**) contents of hazardous substances 5-HMF and 3-DG in different fermented milks. Different lowercase letters indicate statistically significant differences according to Tukey test (*p* < 0.05).

**The contents of lactose, glucose, and hazardous substances in different milks:** Two variants of lactase, namely the low-temperature and medium-temperature lactase, were employed in brown fermented milk processing. To determine the most effective enzyme type, the final products were characterized by lactose and glucose contents. As shown in Figure 1a, the addition of low-temperature lactase decreased the lactose mass ratio 33-fold (from 2.02 to 0.06 g/100 g), and decreased the glucose mass concentration 2.4-fold (from 44.89 to 13.32 g/L), when compared with the commercially fermented milk (the content of lactose and glucose were 2.02 g/100 g and 44.89 g/L, respectively). A similar trend is observed when adding medium-temperature lactase, which led to corresponding 21-fold and 1.3-fold decreases, respectively. In both the cases, the lactose mass ratios were lower than 0.5 g/100 g, meeting the criteria for being lactose-free. Compared with commercial milk, the significant sugar reduction is attributed to the absence of exogenous sugars during our fermentation process. The endogenous sugars which originated from the lactose hydrolysis, mainly glucose, thereby largely took part in the Maillard reaction and were greatly consumed [27].

The hazardous compounds were also examined with 5-Hydroxymethylfurfural (5-HMF) and 3-Deoxyglucosone (3-DG) as the target markers, since they are the compounds generated in the browning of foods and formed via the dehydration and degradation of glucose, respectively (Figure 1b). For the milk using low-temperature lactase, the mass fractions of 5-HMF and 3-DG decreased by 24% and 22%, respectively, while for medium-temperature lactase, similar decreasing trends of 10% and 6% were observed. These results show that our strategy could significantly inhibit the generation of hazardous substances. The possible reason may be associated with the glucose content in different milk [28]. Cardoso et al. [29] found that the generation of 5-HMF and 3-DG is positively related to the glucose level in the Maillard reaction. Since the amount of glucose in commercial products was far more than that in our products, much more hazardous substances were generated. These findings indicate that during the browning process, using lactose hydrolysis instead of adding sugars could largely enhance the health quality of the milk.

**Figure 2:**
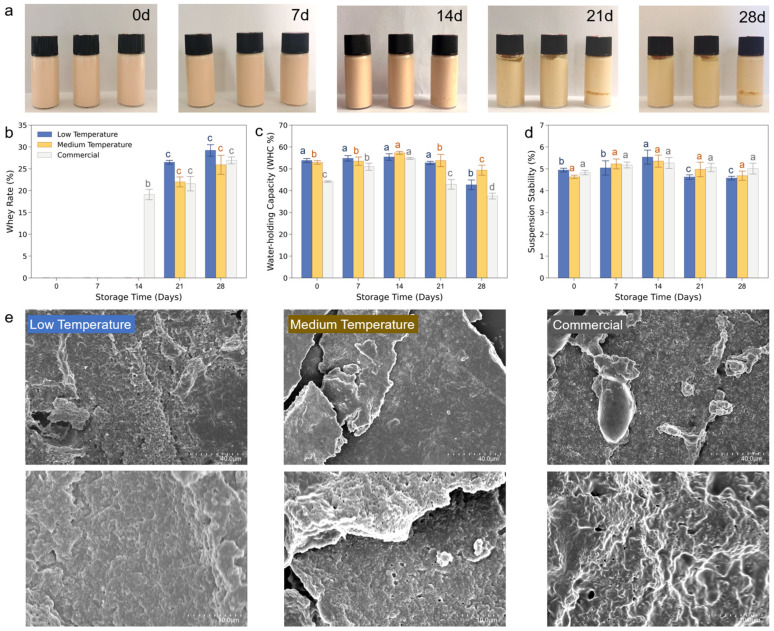
(**a**) The storage of different fermented milks over 0–28 days in photos. The letter “d” indicates storage days. The storage properties were characterized by (**b**) whey rate, (**c**) water-holding capacity (WHC), and (**d**) suspension stability. Different lowercase letters indicate statistically significant differences according to the Tukey test (*p* < 0.05). (**e**) The surface topography of the milks after lyophilization in different scales (the upper part is in 2000× and the lower part is 8000× magnification).

**The storage properties of different milks:** Next, the storage properties of different milk were examined by storing them for 0–28 days, as shown in Figure 2a. During the initial 0–7 days of storage, all the milks exhibited a uniform color, and a smooth texture without bubbles or whey separation. However, as the storage time lengthened, the apparent morphological characteristics of the three groups of fermented milk gradually changed. This was primarily due to the continued fermentation and acid production by the lactic acid bacteria in the milk, which can disrupt the protein network structure and cause whey separation [30]. In general, it can be observed that all three groups of milk exhibited desirable apparent characteristics during the initial 0–7 days of storage. After 14 days of storage, the commercial fermented milk exhibited a slightly higher degree of texture and apparent quality deterioration compared to our homemade fermented milks. Beyond 28 days of storage, all three groups of fermented milk developed off-flavors and became inedible.

To quantify the storage properties, the milks were characterized by three indicators: whey rate, water-holding capacity (WHC), and suspension stability. Figure 2b demonstrates the variance in whey precipitation rate between different fermented milks during storage. On days 7–14, the whey began to precipitate in commercially fermented milk, while our milk remained rather stable. Since day 14, the whey of all the three milks grew significantly and almost reached the same level. The reason is that as storage time passed, the sugar in the base milk is partially converted into lactic acid by the action of various enzymes, originating from the growth of thermophilic lactic acid bacteria. The protein interactions and the gel network structure are severely dissociated under lactic acid [31], resulting in water loss and whey precipitation. This assessment suggests that our milks have a better short-term storage life than commercial milk.

The WHC of the milks were then analyzed over the same storage period; see Figure 2c. During the whole 28 days, the WHC of our milks was higher than the commercial one, indicating a better storage quality over the whole period. In addition, both of the low- and medium-temperature lactase milks reached the highest WHC at day 14, while the low temperature had a higher WHC beforehand, and the medium temperature came from behind afterwards. The initial increase in WHC can on the one hand be attributed to the rigid structure of the protein gel network that held the water inside the grid, and, on the other hand, the enhancement by the exposure of hydrophilic groups of protein during hydrolysis. However, the entire protein network was dissociated upon the accumulation of lactic acid, finally leading to an irreversible decrease in WHC after a long storage time. This assessment suggests that our milks had a better WHC than the commercial one and maintained a good performance in the first 14 days.

The suspension stability over storage is shown in Figure 2d. Similar to the trend in WHC, the suspension stability reached the highest at day 14, followed by a subsequent decrease afterwards. Averaging over the entire storage period, the suspension stability of our and the commercial milk were virtually at the same level. This similarity may be attributed to the exopolysaccharide galactose being decomposed by lactase into glucose and galactose. Kruif and Tuinier [32] reported that complex condensation of protein could occur when the exopolysaccharide content is insufficient to completely envelop the protein. In such cases, one exopolysaccharide chain can bind to the surfaces of multiple proteins and reduce the suspension stability of the system. Therefore, these milks did not present too much difference in this assessment, suggesting that our milk has as good a suspension stability as the milk with commercial maturity.

To show the protein network structure clearly, all the lyophilized samples were further scanned by SEM images to capture their surface topography. As shown in Figure 2e, the milks were formed by the aggregation of casein micelles, resulting in a branched network structure of coarse strands. Compared to the two groups of our homemade fermented milk, the network structure of the commercial fermented milk became uneven, with larger pore spaces. Meanwhile, there was no significant difference between the two groups of homemade fermented milk. These results suggest that our milk has a better texture and quality than the commercial one.

**Figure 3:**
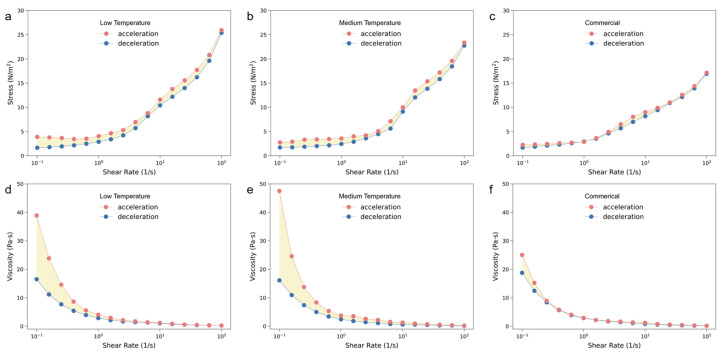
The rheological properties of different fermented milks characterized by (**a**–**c**) stress and (**d**–**f**) viscosity upon shearing. The thixotropic ring area is colored in yellow.

**The rheological characterization of different milks.** Then, the rheological properties were measured to assess the stability of different milks under mechanical conditions, as shown in Figure 3a–c. Hysteresis phenomena can be observed in all the milks, where the shear stress gradually increased with the acceleration process (i.e., increasing of shear rate), and decreased with the deceleration process. As the rate of shear increased, the apparent viscosity of all yogurt samples presented a similar increasing trend (Figure 3d–f), which is consistent with the results of Jiao et al. [33]. At the lower shear rate, the yogurt samples showed an apparent high viscosity. As the shear rate increased, viscosity rapidly reduced, which resulted in a pseudoplastic fluid with a shear-thinning property [34]. The changed rheology was attributed to the destruction of the protein network, the rearrangement of protein micelles, and the occurrence of oil droplets during the shearing process. For our products, the thixotropic ring area of hysteresis (yellow color) was much larger than the commercial one, in which the hysteresis had almost disappeared. That means that much more energy was adsorbed by our yogurt and a longer time was needed until the yogurt recovered during the shearing. This is reasonable because commercially fermented milk usually contains additional stabilizers which result in better structural stability, while the two groups of homemade fermented milk did not have any added stabilizers.

**Figure 4:**
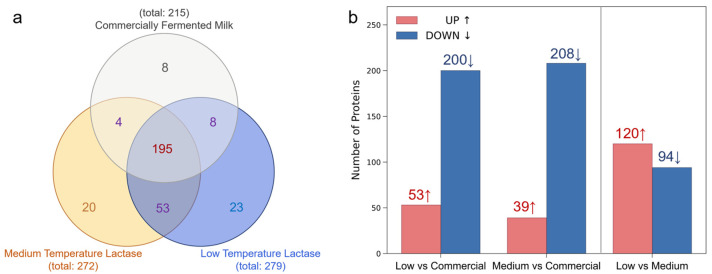
(**a**) Venn diagram of unique and shared proteins in different fermented milks. (**b**) Histogram of quantity distribution of shared proteins in different comparison groups (UP: rate of change >20%; DOWN: rate of change <20%).

**The quantification of proteins in different milks.** Variations in protein composition can have a substantial impact on the nutrition and health of milks. Therefore, we identified and quantified the proteins in different milk samples. As shown in Figure 4a, more than 300 bioactive protein types were identified, while the low- and medium-temperature lactase milks contain 272 and 279 types, much more than the 215 in commercial milk. This may show our milk possibly has more nutritional content, but there is also more uncertainty in this regard. Taking commercial milk as the reference, it was found that in the low-temperature lactase milk, the collagen, β-defensin, hemoglobin, etc., was up-regulated at least 20-fold, while the L-lactate dehydrogenase, serine protease, annexin, etc., was down-regulated almost 100-fold. In the medium-temperature lactase milk, the heat shock protein (70 kDa), collagen β-defensin, hemoglobin, etc., was up-regulated 40-fold, while the phosphatidylethanolamine-binding protein and keratin, etc., was down-regulated almost 100-fold. The possible reason may be that prolonged browning at high temperatures could induce structural damage to milk fat globule membranes and casein micelles, potentially leading to the release of these proteins into the whey fraction and a consequent increase in their abundance. It is also possible that the differences in the origin and processing of milks could lead to the protein variation in products [35].

**Figure 5:**
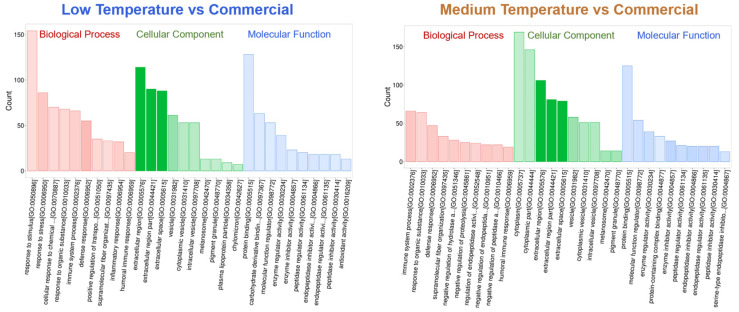
GO analysis of enriched functional categories. Higher transparency of column indicates lower *p*-value and higher confidence.

**Proteomic analyses of different milks.** In order to comprehensively analyze the biological functions of differential proteins in different fermented milks, gene ontology (GO) analysis of the proteins in different comparison groups was carried out to study the enriched functional categories, which were classified into biological processes, cellular components, and molecular functions (Figure 5). Compared with the commercial milk, the main up-regulations of low-temperature lactase milk were responses to stimulus and stress (biological process), extracellular regions and space (cellular component), and protein and carbohydrate-derivative binding (molecular functions). The main up-regulations of medium-temperature lactase milk were correspondingly responses to immune system processes and to organic substances, extracellular regions and space, protein binding, and molecular function regulation [36]. That is, our two products shared similar changes in several categories, and differed largely to the commercial milk.

**Figure 6:**
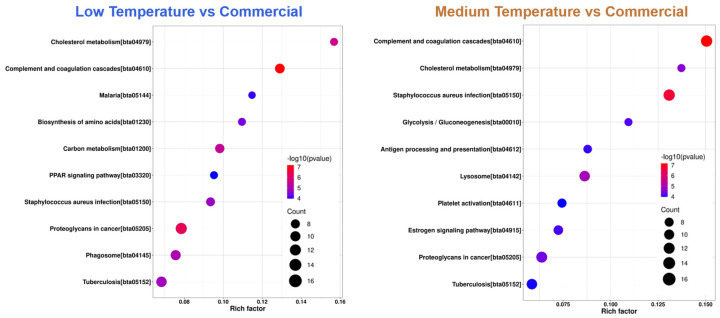
KEGG enrichment pathway analysis. Bigger circle indicates more counts. *p*-value increases from red to blue.

To further explore the differences in biological functions and regulation mechanisms between different milks, we further analyzed the Kyoto Encyclopedia of Genes and Genomes (KEGG) diagrams. In the KEGG enrichment diagrams, the top 10 enrichment channels were selected for mapping, as shown in Figure 6. Compared with the commercial milk, the proteins in low-temperature lactase milk were significantly enriched in proteoglycans in cancer, tuberculosis, and phagosomes, while the proteins in medium-temperature lactase milk were significantly enriched in complement and coagulation cascades, staphylococcus aureus infection, and proteoglycans in cancer. Notably, half of the top 10 channels of the two milks are the same, showing that our two products shared very similar biological functions, with slight differences in concentration.

**Figure 7:**
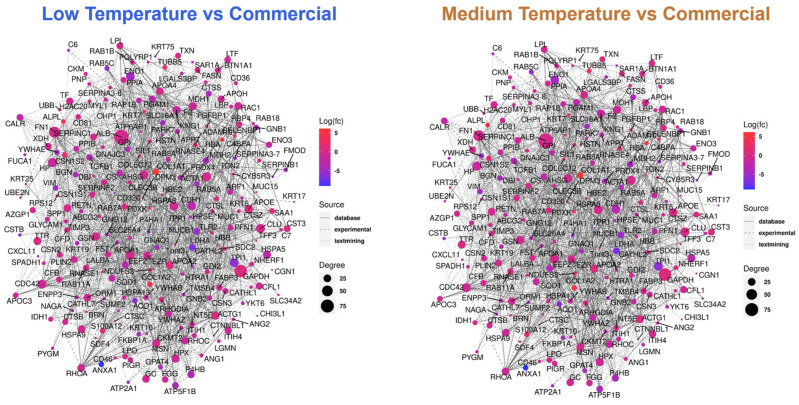
PPI networks analysis. Bigger circle indicates more interactions. Degree of protein regulation decreases from red to blue.

The Protein–Protein Interaction (PPI) networks offer another way to explore the interplay among milk proteins. By mapping these interactions, one can gain valuable insights into the diverse biological processes in which milk proteins participate. In Figure 7, the regulations of protein interaction contents were plotted together to form a complex network. Compared with the commercial milk, the ATP6AP1 and GAPDH are the two proteins which have the greatest number of interactions in both low- and medium-temperature lactase milk. ATP6AP1 is a component of ATPase, which is involved in ATP hydrolysis and energy production. GAPDH is a key enzyme in glycolysis, which is involved in glucose breakdown and also energy production [37]. This suggests the proteins in our products might participate more extensively in cellular energy metabolism compared to commercially fermented milk.

Although proteomics tests have been widely used in evaluating protein components and their nutrition in milk, our study is unique in its comparison of the proteomic profiles produced using different temperature conditions for enzymatic hydrolysis. This approach allows us to directly observe how temperature and enzyme types affects the final protein composition and, by extension, the potential functional properties of the product. The complicated proteomic analyses regarding the biological functions and mechanisms can be highly simplified as follows: Our products may help to resist environmental stress, maintain cell function, and enhance the immune system (from GO); they may enhance adaptability when facing infection and certain diseases like cancer (from KEGG); and may contribute to cellular energy metabolism such as ATP synthesis and glycolysis processes (from PPI). However, one must be aware that this is just a very preliminary inference. The clear picture of the specific beneficial effects of different milks on living organisms must be verified through further extensive physiological experiments. We will continue to explore this theme in the future.

## 4. Conclusions

A novel lactose-free brown fermented milk was produced by employing modern enzymatic hydrolysis technologies, which significantly reduced the lactose and overall sugar content. Compared with commercially fermented milk, the lactose-free milk presented lower quantities of hazardous substances and a better storage stability. Furthermore, the preliminary proteomics analysis showed that our products may help to resist environmental stress, maintain cell function, and enhance the immune system; they may enhance adaptability when facing infection and certain diseases like cancer; and they may contribute to cellular energy metabolism such as ATP synthesis and the glycolysis process. In summary, this experiment provides an important theoretical reference on development and applications in the fermented milk market, but the clear picture of the specific beneficial effects of different milks on living organisms must be verified through further extensive physiological experiments. We will continue to explore this theme in the future.

## Data Availability

The original contributions presented in the study are included in the article, further inquiries can be directed to the corresponding author.
